# CC chemokine receptor 7 promotes macrophage recruitment and induces M2-polarization through CC chemokine ligand 19&21 in oral squamous cell carcinoma

**DOI:** 10.1007/s12672-022-00533-x

**Published:** 2022-07-29

**Authors:** Wan-Hang Zhou, Yao Wang, Cong Yan, Wei-Dong Du, Maged Ali Al-Aroomi, Li Zheng, Shan-Feng Lin, Jia-Xing Gao, Sheng Jiang, Zeng-Xu Wang, Chang-Fu Sun, Fa-Yu Liu

**Affiliations:** Department of Oral Maxillofacial-Head and Neck Surgery, School of Stomatology, China Medical University, Oral Diseases Laboratory of Liaoning, 117 Nanjing North Road, Heping District, Shenyang, 110000 Liaoning China

**Keywords:** CCR7, Macrophage, Recruitment, Polarization, Oral squamous cell carcinoma

## Abstract

**Purpose:**

This study aimed to investigate the impact of CC chemokine receptor 7 (CCR7) on the recruitment and polarization of tumor-associated macrophages (TAMs) in oral squamous cell carcinoma (OSCC).

**Methods:**

We analyzed CCR7 expression pattern, clinicopathological significance, and its association with M2 macrophage infiltration in OSCC by bioinformatic methods. Small interfering RNA (siRNA) was utilized to silence CCR7 in OSCC cells. Conditioned media (CM) was harvested from transfected OSCC cells to establish a co-culture model of THP-1 derived macrophages and OSCC cells. Transwell assay and cell adhesion assay were performed to examine the effect of CCR7 on macrophages recruitment and adhesion. Cytoskeleton was labelled by phalloidin to observe macrophage morphological changes. Moreover, phenotypic alteration of macrophages was measured using quantitative real-time PCR (qRT-PCR), flow cytometry, and immunofluorescence (IF) staining. Ultimately, recombinant human CCL19 and CCL21 were added into the medium of THP-1 derived macrophages to explore their effects on polarization in vitro.

**Results:**

In OSCC patients, the overexpression of CCR7 positively correlated with lymph node metastasis and M2 macrophage infiltration. Macrophage not only exhibited enhanced migration, invasion and adhesion abilities, but also appeared more spindle and branched in vitro when treated with CM from OSCC cells. However, these phenomena were abrogated with knockdown of CCR7. We also discovered that inhibition of CCR7 in OSCC cells suppressed TAMs polarization to an M2 phenotype. In addition, recombinant human CCL19 and CCL21 promoted macrophage M2-polarization in vitro.

**Conclusion:**

CCR7 in OSCC cells promoted recruitment and M2-polarization of THP-1 derived macrophages in vitro by regulating production of CCL19 and CCL21.

**Supplementary Information:**

The online version contains supplementary material available at 10.1007/s12672-022-00533-x.

## Introduction

Head and neck squamous cell carcinoma (HNSCC) is one of the most common cancer worldwide and is responsible for around half a million deaths globally every year [1]. Among the many types of HNSCC, oral squamous cell carcinoma (OSCC) is the most prevalent pathological form [2, 3]. OSCC is a major health threat and one of the leading causes of cancer death in many regions and countries [2–4]. The global incidence of OSCC is predicted to rise by 62% to 856,000 cases by 2035 [2]. Although substantial advances have been made in screening, diagnosis, and multidisciplinary treatment, the mortality of patients with OSCC has remained steadily high over the past few decades [5]. In this context, an in-depth understanding of the OSCC pathogenesis is urgently needed.

Tumor microenvironment (TME) is a delicate dynamic ecosystem containing cellular and non-cellular components that cancer cells depend for sustained growth, invasion, and metastasis [6]. The intricate interplay between tumor cells and surrounding TME cells leads to malignant evolution of oral premalignant lesions and adverse prognosis of OSCC patients [7, 8]. For example, Kujan et al. found significant positive associations between regulatory T cell (Treg)-related proteins (including FOXP3, TGF-β, IL-6, and IL-10) and the histopathological grade of oral epithelial disorders, implying a possible role of tumor-infiltrating immune cells play in evading the immune system and potentially driving malignant transformation [9]. Macrophages are phagocytic and antigen-presenting cells of innate immune system. Mirroring the Th1/Th2 paradigm, macrophages could be divided into two subgroups based on their degree of differentiation and functional capacity, which represent two extreme edges of a continuum of phenotypic range. The classically activated M1-type boosts an antitumor response, while the alternatively activated macrophage that displays anti-inflammatory characteristic is included in the M2 classification [10]. Tumor-associated macrophages (TAMs), which tend to present a M2 phenotype, are among the most abundant and decisive immune cells in the TME [11]. Compelling evidences have proposed that TAMs create an immunosuppressive milieu, exhibit increased pro-tumoral activities and affect virtually all aspects of tumor tissues [12–14]. In OSCC, TAMs have been found particularly higher enriched in the metastatic group than in the non-metastatic and control groups, implying a possibly role in facilitating tumor invasion and metastasis [15]. Similar results were also acquired from several systematic review, showing that elevated concentrations of CD163+ TAMs were associated with worse prognosis, and may serve as one of the most promising predictors of survival in OSCC patients [16–18]. Due to these findings, TAMs have been proposed as a potential therapeutic target in OSCC immunotherapy [19]. It should be noted that versatility and plasticity are hallmarks of macrophages [20]. A diverse array of signals generated from tumor cells could profoundly and differentially influence the polarization of TAMs [21]. Nevertheless, the recruitment processes and functional alterations of macrophages in OSCC are still poorly elucidated. There is, therefore, an imperative need to explore the underlying molecular mechanisms of macrophage differentiation in OSCC.

Chemokines are a group of small, secreted, chemotactic proteins with a multifaceted role in various physiological and pathological events, ranging from thymocyte development and leukocyte migration to lymphoid tissue formation [22, 23]. Based on the structural conservation of both cysteine residues and disulfide bonds, chemokines can be further classified into 4 major groups: CXC, CC, C and CX3C [22]. CC chemokine receptor 7 (CCR7) is one of the most prominent chemokines and belongs to the CC subgroup. It has been reported to abnormally express in diverse tumor types [24] and has been blamed for enhancing tumor migration, epithelial mesenchymal transition (EMT) and cancer stemness through combination with its two CC motif ligands: CC chemokine ligand 19 (CCL19) and CC chemokine ligand 21 (CCL21) [25, 26]. More importantly, we have demonstrated that CCR7 contributes to malignant biological behaviors of OSCC cells through various up- and downstream signaling molecules [27–29]. Whether CCR7 participates TAMs infiltration in OSCC, however, remains highly undetermined. Hence, we investigated the function of CCR7 on macrophage recruitment and polarization in OSCC.

## Materials and methods

### Bioinformatic analysis

Initially, the differential mRNA expression data of CCR7 in diverse human cancers were compared with their matched normal tissues via tumor immune estimation resource 2.0 (TIMER2.0) [30] to outline the expression pattern of CCR7. The full names of tumor abbreviations were supplied in Supplementary Table S1. We also selected 2 datasets (GSE25099 and GSE74530) containing OSCC samples and normal oral tissue from gene expression omnibus (GEO) database [31] to further explore CCR7 expression in OSCC. Differences in CCR7 expression and distribution at the protein level were evaluated utilizing immunohistochemistry staining data of two patients (IDs: 1505 and 298) from the Human Protein Atlas (HPA) database [32]. To analyze the association between CCR7 and clinicopathological features of HNSCC, RNA sequencing (RNA-Seq) data and clinical data of HNSCC patients were retrieved from the cancer genome atlas (TCGA).

Then, we investigate the correlation of CCR7 expression with overall immune infiltration degree in TCGA-HNSCC patients by generating Immunescore using ESTIMATE (Estimation of STromal and Immune cells in MAlignant Tumor tissues using Expression data) algorithm [33]. Furthermore, we explored whether CCR7 expression can affect various immune cell infiltration levels in TCGA-HNSCC patients by extracting data from TIMER [34] and TIMER2.0 web server.

### Reagents and antibodies

Dulbecco’s modified Eagle’s medium (DMEM), Roswell Park Memorial Institute medium (RPMI-1640), reduced serum media Opti-MEM®, β-mercaptoethanol, phosphate buffered saline (PBS), fetal bovine serum (FBS), penicillin and streptomycin were obtained from Gibco (Carlsbad, CA, USA). Phorbol-12-myristate-13 acetate (PMA), paraformaldehyde (PFA) and dimethylsulfoxide (DMSO) were obtained from Sigma-Aldrich (St. Louis, MO, USA). Recombinant human CCL19 (cat No: 300-29B) and CCL21 (cat No: 300-35A) were purchased from PeproTech (Rocky Hill, NJ, USA). Antibodies used for flow cytometry, including human Fc-receptor blocking solution (cat No: 422301) and CD206-PE (cat No: 321105) were from BioLegend (San Diego, CA, USA). A mouse monoclonal anti‑CD163 antibody (cat No: sc‑33715) from Santa Cruz (Dallas, TX, USA) was utilized for immunofluorescence staining. Transfection agent INTERFERin® (cat No: 409-10) was obtained from Polyplus (SA, France). All other chemicals were classified as analytical grade reagents.

### Cell lines and culture conditions

Human acute monocytic leukemia cell line THP-1 was purchased from the Cell Bank/Stem Cell Bank, Chinese Academy of Sciences (Shanghai, China). Two well-characterized CCR7 high-expressed OSCC cell lines, PCI-4B and PCI-37B, were kindly donated by the University of Pittsburgh Cancer Institute (Pittsburgh, PA, USA) [35, 36]. THP-1 monocytes were cultured in RPMI-1640, while PCI-4B and PCI-37B cells were maintained in low glucose DMEM. All cells were supplemented with 10% FBS, 100 U/ml penicillin and 100 µg/ml streptomycin. The medium of THP-1 was complemented with an additional 0.05 mM β-mercaptoethanol. All cells were incubated in a humidified atmosphere with 5% CO_2_ at 37 °C.

### Cell transfection

Small interfering RNA (siRNA) sequence 5′-CTGGTCGTGTTGACCTATA-3′ targeting human CCR7 (siCCR7) and negative control siRNA (siNC) were purchased from Ribobio (Guangzhou, China). The transient transfection was conducted according to the manufacturer’s instructions. In brief, PCI-4B and PCI-37B cells were seeded the day before transfection to reach 30–50% confluency at the time of transfection. siRNA duplexes were diluted into reduced serum media Opti-MEM®. Then, add transfection reagent INTERFERin® into the siRNA solution, vortex-mixed, and incubated for 10 min at room temperature. Finally, INTERFERin®-siRNA complexes were added in complete fresh medium to incubate cells at 37 ℃. 24 h later, the transfected cells were collected for further experiments. The knockdown efficiency of siRNA was tested by quantitative real-time PCR assay.

### Quantitative real-time PCR (qRT-PCR)

Quantitative real-time PCR (qRT-PCR) was carried out to evaluate the mRNA expression levels of CCR7 and macrophages-related genes in OSCC and THP-1 cells. Total RNA was isolated from cultured cells with TRIzol Reagent (TaKaRa, Kyoto, Japan). After examining the concentration and purity using a NanoDrop spectrophotometer (Thermo Scientific, MA, USA), RNA was reversely transcribed into cDNA using the PrimeScript RT reagent Kit (TaKaRa, Kyoto, Japan) according to the protocols recommended by the manufacturer. Subsequently, the cDNA was subjected to qRT-PCR detection using a TB Green® Premix Ex Taq™ II reagent Kit (TaKaRa, Kyoto, Japan) via an ABI QuantStudio3 Real-Time PCR System (Applied Biosystems, CA, USA). Housekeeping gene GADPH was used to normalize relative mRNA levels, calculated by the 2^−ΔΔCT^ method. The primer sequences designed by Sangon Biotech (Shanghai, China) are presented in Table 1.

**Table 1:** Primers used for qRT-PCR

Gene	Forward (5′–3′)	Reverse (5′–3′)
GAPDH	ATCCCATCACCATCTTCC	GAGTCCTTCCACGATACCA
CCR7	TGGTGATCGGCTTTCTGGTC	CACCTTGATGGCCTTGTTGC
CD68	GCTACATGGCGGTGGAGTACAA	ATGATGAGAGGCAGCAAGATGG
CD206	GACGTGGCTGTGGATAAATAAC	CAGAAGACGCATGTAAAGCTAC
CD163	CCTTGGGGTTGTTCTGTTGG	CATGGGAATTTTCTGCAAGCC
IL-10	CCAAGACCCAGACATCAAGG	AAGGCATTCTTCACCTGCTC
TGF-β1	CAAGTGGACATCAACGGGTTC	GCCATGAGAAGCAGGAAAGG

*GAPDH* glyceraldehyde-3-phosphate dehydrogenase; *CCR7* CC chemokine receptor 7; *IL-10* interleukin-10; *TGF-β1* transforming growth factor-β1

### Preparation of conditioned medium

To obtain conditioned media (CM) of OSCC cell lines, PCI-4B and PCI-37B, when up to 80% confluence, were washed by PBS thrice and cultured for an additional 72 h with fresh RPMI-1640 medium without serum. Then, the cell-free supernatants were harvested, centrifuged at 1000 rpm for 5 min and collected as CM after being filtered using 0.22-μm polyvinylidene difluoride membrane filters. CM was aliquoted, stored immediately at − 80 ℃ and avoid repeated freeze/thaws until future experiments.

### Transwell cell migration assay

Transwell cell migration assays were conducted to examine the migration ability of macrophages in response to the CM of OSCC cells. THP-1 was plated on the upper compartment of a 24-well Transwell chamber (8-μm pore diameter; Corning, NY, USA) at 1 × 10^6^ cells/ml density in 200 μl fresh serum-deprived RPMI-1640 medium. Meanwhile, 800 μl CM or fresh RPMI-1640 medium alone as a blank control for a spontaneous migration were added to the lower chamber. After 24 h, the cell suspension in the upper chamber was aspirated. The migrated cells within the lower chamber were photographed by Nikon Eclipse Ts2R inverted fluorescence microscope (Nikon, Tokyo, Japan) and then counted using LUNA-II™ Automated Cell Counter (Logos Biosystems, South Korea) to quantify cell movement.

### Transwell cell invasion assay

Transwell chambers coated with Matrigel matrix (Corning, NY, USA) were utilized to perform Transwell cell invasion assays. THP-1 cells were seeded in the upper chamber with 200 μl serum-free RPMI-1640 medium at a density of 1 × 10^6^ cells/ml. Then, 800 μl CM or fresh RPMI-1640 medium alone as a blank control for spontaneous invasion were added to the lower chamber. After incubation for 48 h, the numbers of invaded THP-1 cells within the lower chamber were photographed by Nikon Eclipse Ts2R inverted fluorescence microscope and counted by LUNA-II™ Automated Cell Counter.

### Cell adhesion assay

Briefly, THP-1 cells were suspended in the CM or fresh RPMI-1640 medium alone as a blank control for spontaneous adhesion and seeded in the 24-well plate at the density of 2 × 10^4^ cells/well. After 24 h, the wells were gently washed with PBS to remove non-adhered cells. The adherent THP-1 cells were then fixed with 4% PFA for 15 min and stained using DAPI (Beyotime, Shanghai, China) for 10 min at room temperature. Adherent cells were observed under Nikon Eclipse Ts2R inverted fluorescence microscope and counted using ImageJ software (National Institutes of Health, Bethesda, MD, USA).

### Macrophage differentiation and co-culture system

THP-1 monocytes were differentiated into macrophages as previously described [37]. In brief, the THP-1 cells were seeded into 6-well culture plates at 1 × 10^6^ cells/ml in the presence of 100 ng/ml PMA for 24 h to obtain resting macrophages (M0). Subsequently, PMA was removed, and M0 cells were cultured with fresh RPMI-1640 medium for another 48 h to allow cellular recovery. To establish a co-culture system, the M0 cells were cultured in the CM of OSCC cell lines and harvested for further analysis after 72 h. CCL19 or CCL21 at 400 ng/ml was added into the FBS-free RPMI-1640 medium for 72 h to explore their effects on macrophage polarization in vitro.

### Macrophage morphology and imaging

In order to investigate the effect of OSCC CM on macrophage morphology, co-cultured macrophages were fixed in 4% PFA and permeabilized by 0.2% Triton X-100 (Beyotime, Shanghai, China) beforehand. Actin-Tracker Red-555 Fluorescent Phalloidin (dilution 1:100, Beyotime, Shanghai, China) was applied to label the cytoskeleton, and DAPI (Beyotime, Shanghai, China) was employed to stain the nuclei. Fluorescently labelled cells were then observed and photographed under Nikon Eclipse Ts2R inverted fluorescence microscope.

### Flow cytometry

Flow cytometry was performed to detect cytomembrane CD206 (also known as mannose receptor c-type 1, MRCI) expression of macrophage after co-cultured with CM. M0 macrophages without any further treatment were set up as the blank control group. Macrophages were washed, scraped off using cell scrapers and resuspended in ice-cold PBS containing 2% FBS. Human Fc-receptor block was used to reduce non-specific immunofluorescent staining. Next, cells were labelled with PE-conjugated mouse anti-human CD206 for 30 min at 4 °C in dark. At last, the proportion of CD206+ macrophages was analyzed by a BD FACSCanto II flow cytometer (BD Biosciences, San Jose, CA, USA) (10,000 events were recorded for each sample).

### Immunofluorescence (IF) staining

Immunofluorescence (IF) staining was carried out to observe the CD163 expression level of CM-treated macrophages. After discarding culture medium, macrophages growing on coverslips were collected and washed with PBS containing 2% FBS for 3 times. Then, cells were fixed with 4% PFA at room temperature for 15 min and permeabilized by 0.2% Triton X-100 for 10 min. Unspecific binding sites were later blocked by QuickBlock™ Blocking Buffer (Beyotime, Shanghai, China) for 1 h at room temperature. The cells were incubated with the primary CD163 antibody conjugated to Alexa Fluor® 488 (dilution 1:200) at 4 °C in dark overnight. Then, samples were stained with DAPI for 10 min to label the nuclei. Finally, cells were observed and photographed by Nikon Eclipse Ts2R inverted fluorescence microscope.

### Statistical analyses

All statistical analyses were conducted using GraphPad Prism 9 (GraphPad Software, Inc., CA, USA). The results were presented as the mean ± standard deviation (SD) of at least three different experiments. The significant difference between two groups was determined by independent sample or paired-sample Student's t test. The one-way analysis of variance (ANOVA) followed by the Dunnett's multiple comparisons test was employed to compare more than two groups. Spearman correlation analysis was applied to assess the statistical significance of correlations between two variables. Flow cytometry data analysis was performed using Flowjo software (Tree Star, Ashland, OR, USA). A two-tailed *P* < 0.05 was considered statistically significant if not especially noted.

## Results

### Overexpression of CCR7 positively correlated with lymph node metastasis and M2 macrophage infiltration in OSCC patients

**Fig. 1:**
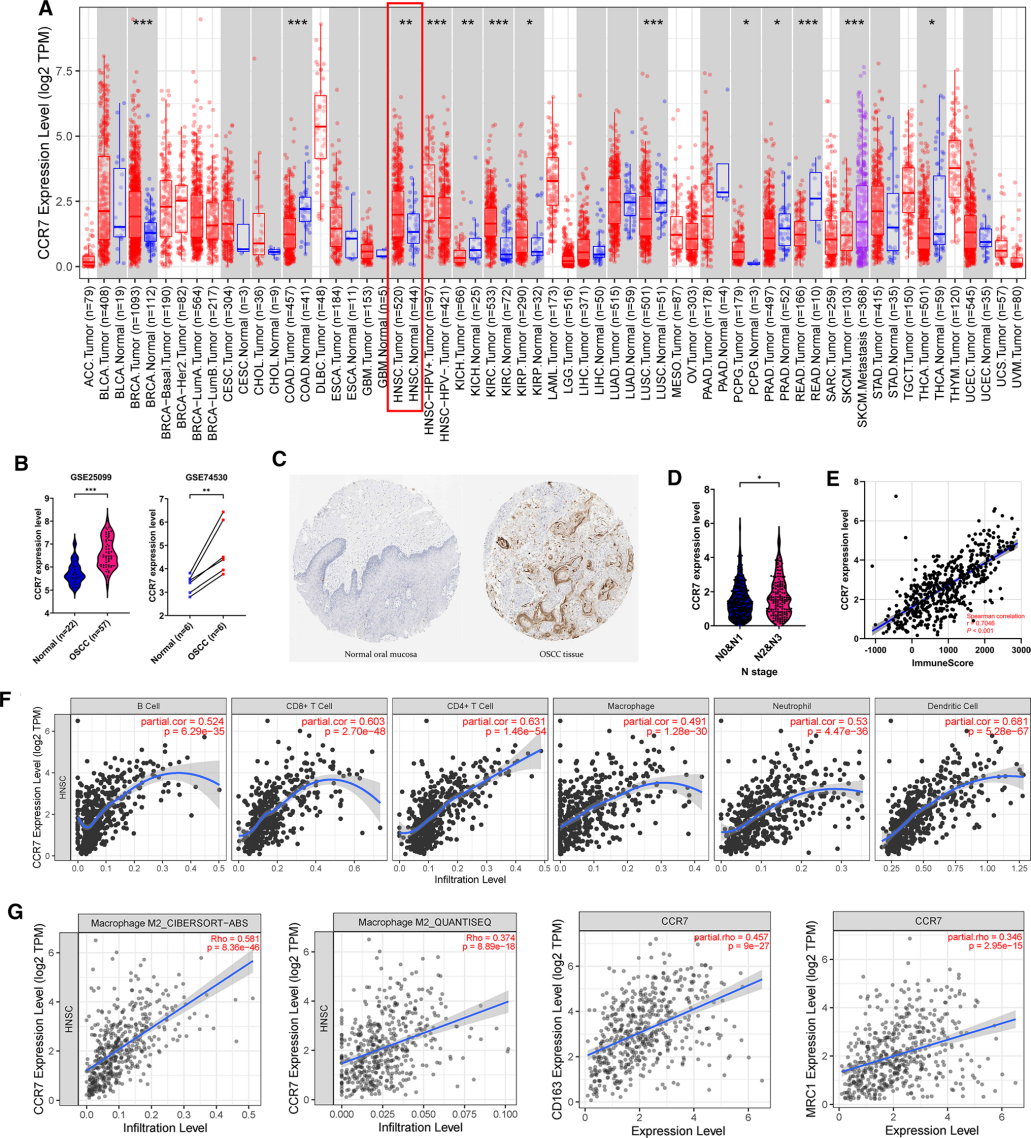
CCR7 is overexpressed and positively correlated with M2 macrophage infiltration in OSCC patients. **A** CCR7 expression patterns across different tumour types in TIMER2.0. The red box represents HNSCC tissues and adjacent normal tissues. **B** CCR7 expression levels in OSCC tissues and normal oral tissues in GSE25099 and GSE74530 dataset. **C** CCR7 protein expression levels in OSCC tissues and normal oral epithelial tissues, determined by immunohistochemistry staining from HPA website. magnification, × 4. **D** Relationship between CCR7 expression and N stage of HNSCC patients, based on TCGA. **E** The association between CCR7 expression and the Immunescore of TCGA-HNSCC patients generated by ESTIMATE algorithm. **F** Correlation between CCR7 expression level and immune infiltration levels in TCGA-HNSCC patients based on TIMER2.0 database. **G** Correlation between CCR7 expression and M2 macrophage in TCGA-HNSCC patients, based on TIMER2.0 database. *P < 0.05, **P < 0.01, ***P < 0.001

In order to have a broad view of CCR7 expression pattern in pan-cancer scale, we downloaded RNAseq data of TCGA tumors from TIMER2.0 database. The results revealed that CCR7 was notably up-regulated in various tumor types, including HNSCC (red box) (Fig. 1A). Two GEO datasets were applied to further estimate CCR7 expression level in OSCC. We found that CCR7 was overexpressed in OSCC, whether viewed from the overall sample or the matched sample of a single patient (Fig. 1B). This generally in accordance with the immunohistochemical data obtained from HPA database, which showed that CCR7 was almost not expressed in normal oral epithelial tissue, while OSCC tissue had strong staining (Fig. 1C). Clinical information retrieved from TCGA-HNSC cohort was then employed to examine the clinicopathological significance of CCR7. As displayed in Fig. 1D, patients with severe lymph node involvement are more likely to overexpress CCR7 (*P* < 0.05).

Next, we investigated the latent role CCR7 plays in the tumor immunology of HNSCC. ESTIMATE algorithm demonstrated an obvious positive correlation between CCR7 and Immunescore in HNSCC patients (Fig. 1E, Spearman correlation coefficient = 0.7046, *P* < 0.001). Similarly, data from TIMER web server suggested that CCR7 had a noticeable effect on almost all types of immune cells in HNSCC (Fig. 1F). In view of the widely-discussed pro-tumor functions of M2 macrophages, we thus examined the association between CCR7 and M2 macrophages. Of note, 2 different algorithms (CIBERSOFT-ABS and QUANTISEQ) indicated that CCR7 considerably associated with M2 macrophage infiltration level in HNSCC (Fig. 1G; CIBERSOFT-ABS Spearman correlation coefficient = 0.581, *P* = 8.36e−46; QUANTISEQ Spearman correlation coefficient = 0.374, *P* = 8.89e−18). Meanwhile, spearman correlation analysis illustrated a strong correlation between CCR7 and two essential M2 macrophage biomarkers, CD163 and MRC1 (CD206), in HNSCC patients (Fig. 1G; CD163 Spearman correlation coefficient = 0.457, *P* = 9e−27; CD206 Spearman correlation coefficient = 0.346, *P* = 2.95e−15). The above results demonstrated that CCR7 is significantly up-regulated in OSCC patients and contributes to lymph node metastasis and M2 macrophage infiltration.

### CCR7 deficiency in OSCC cells restrained macrophage recruitment and adhesion

**Fig. 2:**
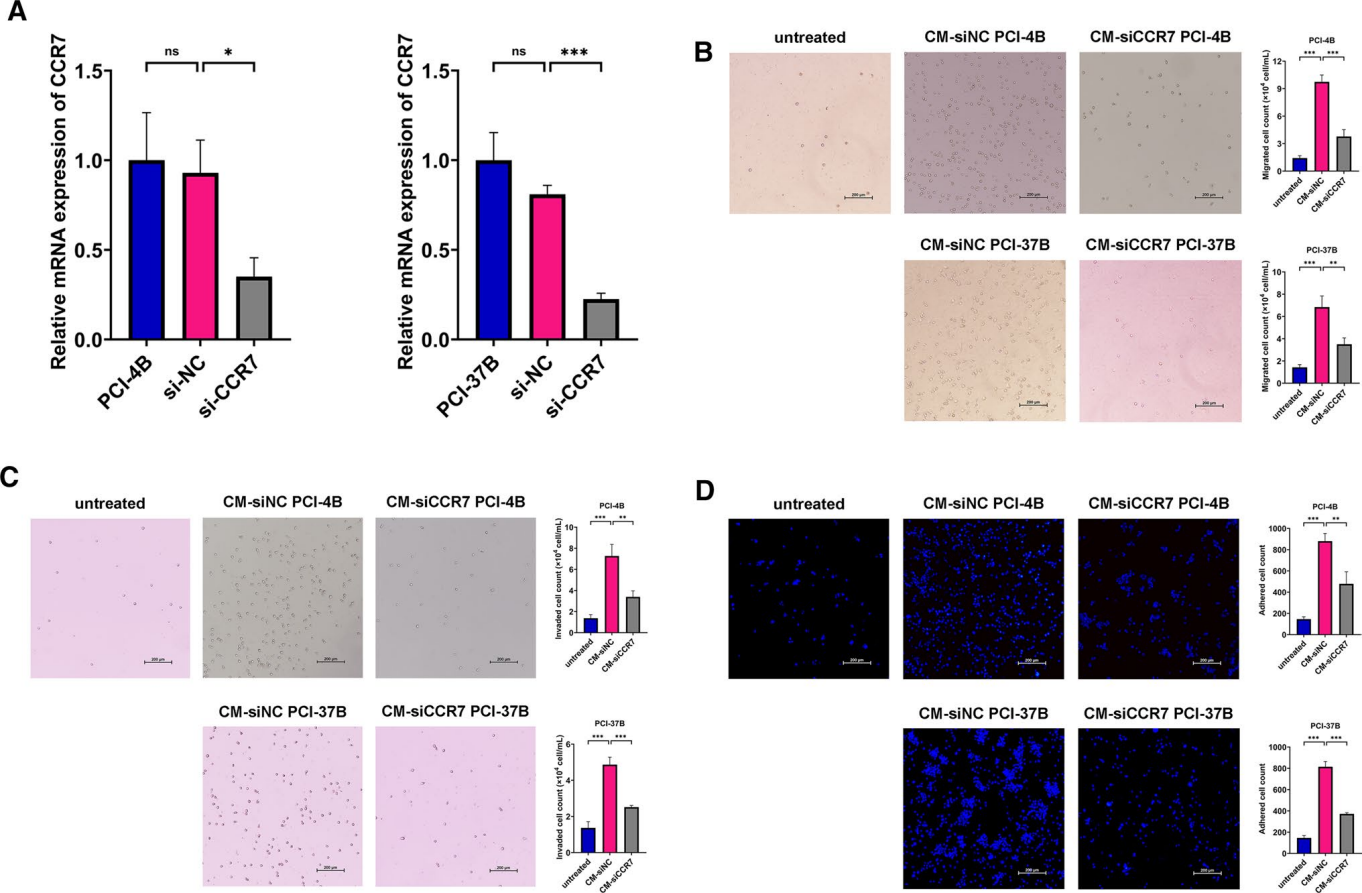
CCR7 deficiency in OSCC cells restrained macrophage recruitment and adhesion. **A** qRT-PCR examining CCR7 knockdown efficiency in OSCC cell lines PCI-4B and PCI-37B. **B** The effects of OSCC CM on the migration ability of THP-1 cells were quantified by Transwell cell migration assay. Representative images and statistical analysis histograms were shown (scale bar: 200 μm). **C** The effects of OSCC CM on the invasion ability of THP-1 cells were estimated by Transwell cell invasion assay. Representative images and statistical analysis histograms were shown (scale bar: 200 μm). **D** The effects of OSCC CM on the adhesion of THP-1 cells assessed by cell adhesion assay. The nuclei were labelled with DAPI (blue). Representative images and statistical analysis histograms were shown (scale bar: 200um). The results are presented as the mean ± SD, *ns* not significant, *P < 0.05, **P < 0.01, ***P < 0.001

To uncover the potential function of CCR7 regulating macrophage in OSCC, siCCR7 and siNC were utilized to transfect PCI-4B and PCI-37B cells. The results of qRT-PCR confirmed substantial CCR7 downregulation at mRNA levels 24 h post transfection (Fig. 2A). CM was collected as previously described from siNC transfected-OSCC cells (CM-siNC) and CCR7-silenced OSCC cells (CM-siCCR7), respectively. Then, we examined the migration and invasion abilities of macrophages after co-culture with CM from OSCC cells with different CCR7 expression levels. A sketch for the Transwell cell migration and invasion assay was supplied in Supplementary Fig. S1. Compared with blank control, we found that CM-siNC could significantly induce THP-1 cell migration. The CCR7 depletion, however, reverses this trend (Fig. 2B). Invasion assay exhibited a similar pattern (Fig. 2C). In line with our results, TCGA data pointed out that the mRNA expression level of CCR7 is positively related to CD68, a pan-marker of macrophages (Supplementary Fig. S2, Spearman correlation coefficient = 0.289, *P* = 6.27e−11). Moreover, THP-1 cells without any treatment barely attach to the dishes since they grow in suspension. We observed a dramatic increase of adherent THP-1 cells in the CM-siNC group while a reduction in the CM-siCCR7 treatment group. Collectively, these results indicated that CCR7 deficiency in OSCC cells confined macrophage accumulation and adhesion in the TME.

### Knockdown of CCR7 in OSCC cells suppressed macrophage M2-polarization

**Fig. 3:**
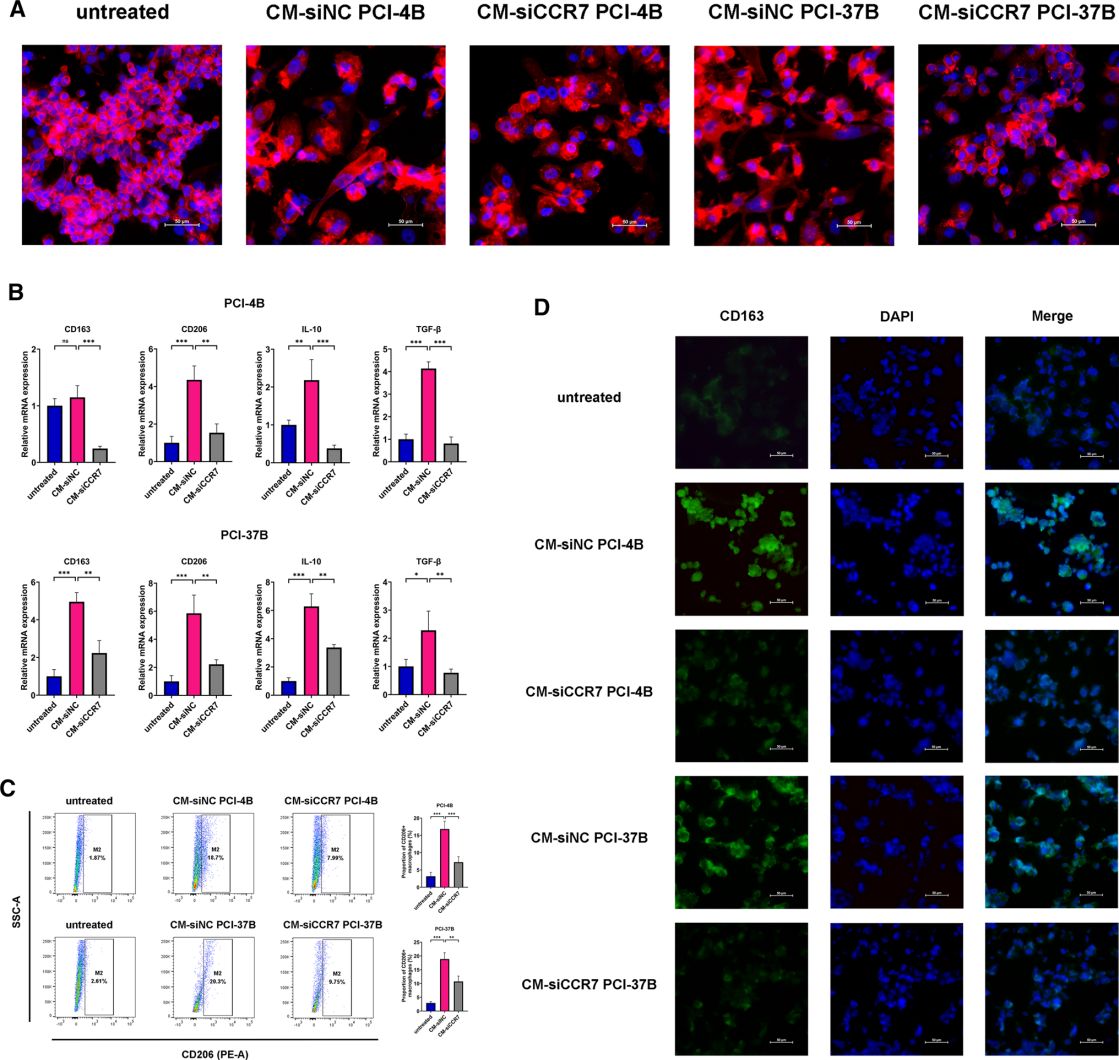
Knockdown of CCR7 in OSCC cells suppressed macrophage M2-polarization. **A** Representative morphological images of THP-1 derived macrophages cultivated with OSCC CM. The cytoskeleton was labelled with Actin-Tracker Red-555 Fluorescent Phalloidin (red), and nuclei were stained with DAPI (blue) (scale bar: 50 μm). **B** The mRNA expression level of CD163, CD206, IL-10 and TGF-β1 of macrophages co-cultured with OSCC CM, evaluated by qRT-PCR. **C** Representative Scatter plots showed the CD206 expression level of macrophages incubated with OSCC CM, measured by flow cytometry. The histogram displayed statistical analysis for the proportion of CD206+ macrophages. **D** Representative fluorescent images of macrophages treated with OSCC CM. The cytomembrane CD163 was labelled with Alexa Fluor® 488 (green), and nuclei were stained with DAPI (blue) (scale bar: 50um). The results are presented as the mean ± SD, ns: not significant, *P < 0.05, **P < 0.01, ***P < 0.001

To further determine whether CCR7 was involved in the process of macrophage activation and polarization in OSCC, we induced suspension THP-1 monocytes into adherent M0 macrophages using PMA. The result of qRT-PCR showed that the mRNA level of pan macrophage marker CD68 was upregulated in M0 macrophages compared with THP-1 cells, suggesting a satisfying induction efficiency (Supplementary Fig. S3, *P* < 0.01). Subsequently, the M0 macrophages were cultivated with CM-siNC and CM-siCCR7 for 72 h, respectively. A sketch for the co-culture system was shown in Supplementary Fig. S4. Through the cytoskeleton staining, we observed that macrophages differ substantially in their morphologies when cultivating with CM with different CCR7 expression levels. Compared to untreated cells, macrophages tended to be larger and behaved more spindle and branched when treated with CM-siNC. Nevertheless, the macrophages were not only smaller but also appeared round and smooth in response to incubation with CM-siCCR7, similar to M0 macrophages (Fig. 3A). On the other hand, according to Fig. 3B, the mRNA levels of M2 macrophage markers CD163, CD206, interleukin-10 (IL-10) and transforming growth factor-β1 (TGF-β1) were upregulated in the CM-siNC groups compared with the untreated M0 group. By contrast, after treatment with CM-siCCR7, the mRNA levels of the above markers were markedly downregulated. We also employed flow cytometry to quantify the membrane expression of CD206. We noticed that nearly 20% of macrophages displayed positive staining of CD206 when coculturing with CM-siNC, whereas the frequency of CD206+ macrophages significantly decreased in CM-siCCR7 the group (Fig. 3C). Consistently, IF staining analysis revealed that the fluorescence intensity of CD163 was considerably higher in the CM-siNC group than in the blank control group and CM-siCCR7 group (Fig. 3D). Combined, these results presented evidence that knockdown of CCR7 in OSCC cells inhibited TAMs polarization to an M2 phenotype.

### CCL19 and CCL21 promoted macrophage M2-polarization in vitro

**Fig. 4:**
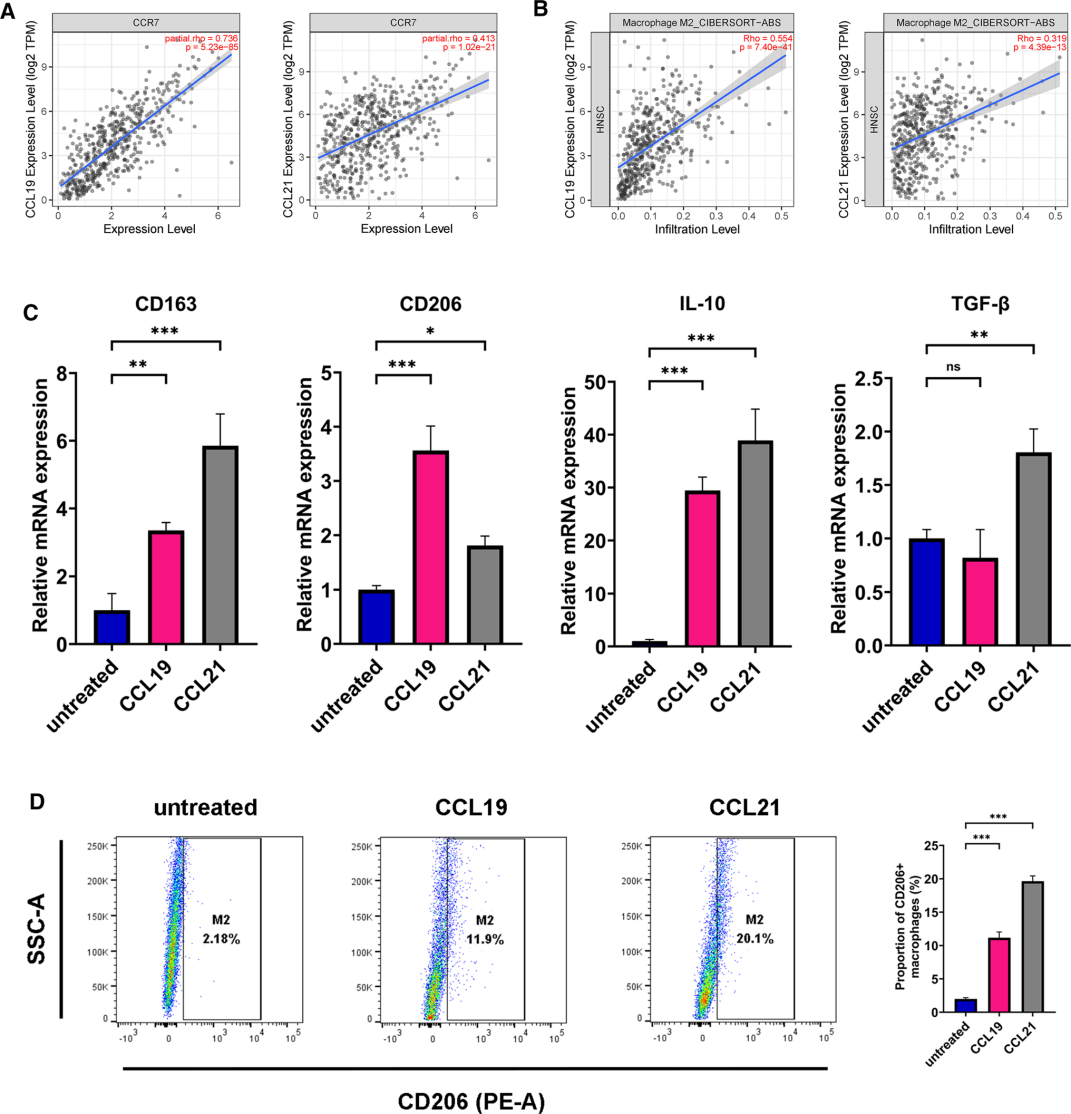
CCL19 and CCL21 promoted macrophage M2-polarization in vitro. **A** Spearman correlation analysis of CCR7 expression with CCL19 and CCL21, based on TIMER2.0 database. **B** Correlation between CCR7 expression and M2 macrophage, based on TIMER2.0 database. **C** The mRNA expression level of CD163, CD206, IL-10 and TGF-β1 of THP-1 derived macrophages stimulated by 400 ng/ml recombinant human CCL19 and CCL21, evaluated by qRT-PCR. **D** Representative Scatter plots showed the CD206 expression level of macrophages incubated with 400 ng/ml recombinant human CCL19 and CCL21, measured by flow cytometry. The histogram displayed statistical analysis for the proportion of CD206+ macrophages. The results are presented as the mean ± SD, *P < 0.05, **P < 0.01, ***P < 0.001

CCL19 and CCL21 are two primary functional ligands for CCR7 [38]. The results based on the TCGA database displayed a positively correlation between CCR7 and both CCL19 and CCL21 in OSCC (Fig. 4A, CCL19 Spearman correlation coefficient = 0.736, *P* = 5.23e−85; CCL21 Spearman correlation coefficient = 0.413, *P* = 1.02e−21). Previous scholars have proved that they can be massively secreted by OSCC cell lines PCI-4B and PCI-37B [39]. What is more, data from TIMER2.0 showed that both CCL19 and CCL21 correlated with macrophage M2-polarization (Fig. 4B, CCL19 Spearman correlation coefficient = 0.554, *P* = 7.40e−41; CCL21 Spearman correlation coefficient = 0.319, *P* = 4.39e−13). Hence, we supposed that CCL19 and CCL21 were involved in M2 polarization of macrophages. In vitro experiments were then carried out to identify the effects of recombinant human CCL19 and CCL21 on THP-1 derived macrophages. The results suggested that CCL19 and CCL21 (both at 400 ng/ml) enhanced mRNA expression level of various M2 markers in vitro (Fig. 4C). Also, the treatment of CCL19&21 significantly increased the proportion of CD206+ macrophages (Fig. 4D). Overall, current evidence seemed to support that CCL19 and CCL21 promoted M2-polarization of macrophages in vitro.

## Discussion

Recent studies support the notion that cancer fate, including malignant progression, intravasation and therapy resistance, results not only from inner genetic changes of tumor cell itself but also from the complicated communication networks that it establishes with surrounding TME [40, 41]. The emerging treatment modality of immunotherapy has been developed, targeting TME to enhance the immune response to tumors and limit the growth of cancer cells [42]. The TME of OSCC is highly immunosuppressive and consists of many different subsets of cells [43], in which TAMs constitute a principal component [15]. It is considered that M2-like TAMs favor tumor growth via inducing tissue remodeling, suppressing adaptive immunity by T-cells, and producing a battery of bioactive molecules [44, 45]. Despite numerous studies regarding this essential topic, the molecular mechanism by which TAMs were recruited and activated is still far from clear. CCR7 is a G protein‐coupled transmembrane chemokine with multiple functions [46]. Our previous studies have verified that CCR7 is associated with the invasion and metastasis of tumors through eliciting a series of intracellular signaling pathways in OSCC [47, 48]. Nonetheless, little is known about the clinicopathological significance of CCR7 in TAMs infiltration. By establishing a co-culture system, our data support a hypothesis that OSCC cells actively recruit peripheral monocytes into TME and promote their polarization towards M2 macrophages through the overexpression of CCR7. Regulation of TAMs is an essential means in the immunotherapy of OSCC patients. Based on current results, depleting TAMs or suppressing their polarization from an M1 to an M2 phenotype by targeting CCR7 may be a novel therapeutic approach and deserves further clinical verification in real world study.

Previous studies postulated that CCR7 is abnormally upregulated in a number of malignancies such as breast cancer [49], lung adenocarcinoma [50] and gastric cancer [51]. In this work, we demonstrated that CCR7 was overexpressed in OSCC. The protein expression data obtained from HPA was generally consistent. Moreover, patients with advanced stages of OSCC are more likely to overexpress CCR7. Shang et al. [52] also discovered that CCR7 expression was significantly higher in OSCC patients with lymph node metastasis compared with those without (*P* = 0.015) and was also associated with tumor size (*P* = 0.014) and clinical stage (*P* = 0.009). These results indicated that CCR7 might serve as an oncogene in OSCC. Given that CCR7 is known to mediate many events in adaptive immune system [53], we next examined the relationship between CCR7 and immune infiltration level in OSCC. ESTIMATE algorithm and TIMER web server revealed that CCR7 expression level was positively associated with increased immune infiltration level in OSCC. Previous studies focused more on its distinct role on regulating T cells homing, activation and recruitment [54, 55]. The biological link between CCR7 and TAMs in OSCC is yet to be illustrated so far. The tumor-infiltrating immune cell models in TIMER2.0 database depicted that the expression of CCR7 promotes M2 macrophages infiltration level and correlates with M2 biomarkers in OSCC. Thereby, it is reasonable to speculate that CCR7 facilitate the malignant process of OSCC by modulating macrophage.

TAMs in the TME are mainly derived from peripheral blood monocytic precursors [44]. Therefore, recruitment of circulating monocytes is of vital importance in the inflammatory and immune responses of TME. Cytokines documented to have chemotactic effects for monocytes include CCL2 [56], CCL15 [57], CCL20 [58], vascular endothelial growth factor (VEGF) [59] and platelet-derived growth factor (PDGF) [60]. When treated with CM-siNC, we noticed an obvious increase in the migrated and invaded THP-1 cells, while this trend was reversed after silencing CCR7. Likewise, Yang et al. reported CCR7 is positively correlated with macrophage migration [61]. It is worth mentioning that CM-siNC enhanced adhesion of THP-1 cells compared to untreated cells, but this phenomenon was less seen in the CM-siCCR7 group. The THP-1 monocytic cell line grows in suspension and does not attach to cell culture surfaces in most cases. Adherence is considered a feature of its maturity, signaling that monocytes have started the differentiation into macrophages [62]. In other words, CCR7 contributed to TAMs accumulation in the TME of OSCC and may induce activation.

It is well acknowledged that macrophages are a rather dynamic cell population, exhibiting remarkable phenotypic heterogeneity and functional plasticity in response to exposure of TME stimuli [21]. We thus established a co-culture system to study the role CCR7 plays in the interaction between macrophages and OSCC cells. Our data suggested that CM-siNC activated macrophages to become M2-like phenotype, whereas a substantial decrease in the expression of M2 macrophage markers (CD163, CD206, IL-10, TGF-β1) was observed after co-cultured with CM-siCCR7. Results from flow cytometry showed that CM-siCCR7 reduced the proportion of CD206+ macrophages in vitro. IF staining data also implied that compared to untreated group, the fluorescence intensity of CD163 was dramatically higher in CM-siNC group, while it barely changed in CM-siCCR7 group. Sunil et al. yielded a similar result, who indicated that compared with wild-type, CCR7-knockout mice presented significantly lower levels of two well-acknowledged M2 macrophage markers (IL-10 and TGF-β1) [63]. In brief, these results reminded us that CCR7 might be an inducer of M2-like TAMs polarization in OSCC.

CCL19 and CCL21 are two natural ligands of CCR7 that widely exist in lymphoid organs and immune cells [46], and have been validated to be produced in large quantities by CCR7 high-expressed OSCC cell lines PCI-4B and PCI-37B [39]. Several recent studies have pointed to a strong association between CCL19&21 and cancer progression [64, 65]. In fact, earlier researchers have highlighted the indispensable roles CCL19 and CCL21 played in attracting TAMs. Cai et al. revealed that CCL19 and CCL21 treatment significantly increased the migration of monocytes, whereas the addition of CCR7-neutralizing antibody abolished both CCL19- and CCL21-induced monocyte migration [66]. Allaire et al. found that in monocytes, CCL19 binding to CCR7 results in potent phosphorylation of MAPK and leads to monocyte migration [67]. Similarly, Ato et al. reported that CCL19 and CCL21 stimulated macrophage migration in vitro in a dose-dependent fashion [68]. More importantly, results from TIMER2.0 database reminded us there is strong relationship between CCL19&21 and M2 macrophages in HNSCC. Accordingly, we conjectured they were involved in the M2 polarization of macrophages. As expected, recombinant human CCL19 and CCL21 largely reeducated M0 macrophage to M2 phenotype in vitro. Our results were corroborated by an in vivo study by Shields et al. [69]. They used short hairpin RNA (shRNA) to knockdown endogenous CCL21 secretion in murine melanoma cells and found that CCL21^low^ tumors contained lower amounts of TGF-β1. TGF-β1 is not only a known M2 macrophage marker but also a critical immunomodulator that can shift the macrophage populations from M1 to pro-tumoral M2 phenotype [70]. Researchers also added CCL19 directly to cultures of human peripheral blood mononuclear cell (PBMC), and observed a significant enhancement of M2 marker IL-10 production [71]. In agreement with this, Pickens et al. demonstrated that CCL19 and CCL21 induce secretion of proangiogenic factors from macrophages [72], which is among the most crucial characteristics of M2 macrophages. Taken together, the present study implied that OSCC cells could educate TAMs toward M2 phenotype that promotes tumor growth and spread via, at least in part, CCR7-CCL19&21 axis.

CCL19&21 binding to CCR7 causes conformational changes, triggers various signaling cascades and elicit their biologic functions [73]. Our previous studies have uncovered that CCL19 could induce phosphorylation of JAK2/STAT3 and NF-κB, activate MAPK and PI3K/Akt signaling pathway through interaction with CCR7 [47, 48, 74, 75]. Similarly, Chen et al. [26] found that CCR7/CCL21 axis activated the JAK2/STAT3 signaling pathway. Many investigators have reported that the acquisition of TAM M2 phenotype is closely associated with pathways mentioned above, especially NF-κB [37, 76–78]. Hence, it is possible that the interaction of CCR7 and CCL19 or CCL21 promotes macrophage M2 polarization via one of these signaling pathways. Although the underlying mechanism is unable to be clarified by now, this will be the focus to investigate in our follow-up studies.

Nevertheless, it is undeniable that several potential limitations should be taken into consideration when interpreting our results. Firstly, since we focused on the biological functions of CCR7, two CCR7 high-expressed OSCC cell lines, PCI-4B and PCI-37B, were applied in this study. More types of tumor cell lines should be used in future analysis. Secondly, despite THP-1 cell line resembles native macrophages in morphological and functional properties and is commonly used as an appropriate model to imitate TAMs differentiation in vitro [79], some argue that heterogeneity still exists between THP-1 cells and primary cells [80]. Consequently, additional investigations using primary human macrophages are warranted to reach a more convincing conclusion. Last but not least, our findings should be further verified in a suitable immunocompetent OSCC animal model.

## Conclusion

In conclusion, our data demonstrated that CCR7 was up-regulated in OSCC patients and correlated with increased M2 macrophage infiltration levels. Further in vitro investigation favored the assumption that CCR7 in OSCC cells promoted recruitment and M2-polarization of THP-1 derived macrophages, which was mainly modulated by CCL19 and CCL21. Although it requires further clinical verification, our work provided a potential CCR7-based antitumor strategy for OSCC patients.

## Supplementary Information

Below is the link to the electronic supplementary material.

Supplementary file1 (PDF 467 kb)

## Data Availability

The datasets generated during and/or analyzed during the current study are available in the TIMER2.0 repository, http://timer.comp-genomics.org/; GEO repository, https://www.ncbi.nlm.nih.gov/geo/; HPA repository, https://www.proteinatlas.org/; TCGA repository, https://tcgadata.nci.nih.gov/tcga; ESTIMATE repository, https://sourceforge.net/projects/estimateproject/; TIMER repository, https://cistrome.shinyapps.io/timer/**.**
