# Supplementary material for: CC chemokine receptor 7 promotes macrophage recruitment and induces M2-polarization through CC chemokine ligand 19&21 in oral squamous cell carcinoma
Source: Discov Oncol. 2022 Jul 29;13:67. doi: 10.1007/s12672-022-00533-x (PMC9338204; doi:10.1007/s12672-022-00533-x)

Discover Oncology

Appendix to the manuscript

CC chemokine receptor 7 promotes macrophage recruitment and induces M2-polarization through CC chemokine ligand 19&21 in oral squamous cell carcinoma

Wan-Hang Zhou, Yao Wang, Cong Yan, Wei-Dong Du, Maged Ali Al-Aroomi, Li Zheng, Shan-Feng Lin, Jia-Xing Gao, Sheng Jiang, Zeng-Xu Wang, Chang-Fu Sun, Fa-Yu Liu*

Department of Oral Maxillofacial-Head and Neck Surgery, School of Stomatology, China Medical University; Oral Diseases Laboratory of Liaoning, Shenyang 110000, China

***Corresponding author:**
Fa-Yu Liu
Department of Oral Maxillofacial-Head and Neck Surgery, School of Stomatology, China Medical University; Oral Diseases Laboratory of Liaoning
117 Nanjing North Road, Heping District, Shenyang, Liaoning 110000, China
Tel: +86 24 22894773
Fax: +86 24 86602310
E-mail: lfyhjk@126.com

Table of contents

1. The full names of tumor abbreviation from TCGA (Table S1) 3

2. A sketch for the Transwell assay (Figure S1) 5

3. Correlation between CCR7 and CD68 (Figure S2) 6

4. mRNA expression level of CD68 in M0 macrophages (Figure S3) 7

5. A sketch for the M0 macrophages and OSCC cells coculture system (Figure S4) 8

1. Table S1. The full names of tumor abbreviation from TCGA.

Table S1. The full names of tumor abbreviation from TCGA

Abbreviations	Tumor full manes
ACC	Adrenocortical carcinoma
BLCA	Bladder Urothelial Carcinoma
BRCA	Breast invasive carcinoma
CESC	Cervical squamous cell carcinoma and endocervical adenocarcinoma
CHOL	Cholangiocarcinoma
COAD	Colon adenocarcinoma
DLBC	Lymphoid Neoplasm Diffuse Large B-cell Lymphoma
ESCA	Esophageal carcinoma
GBM	Glioblastoma multiforme
HNSC	Head and Neck squamous cell carcinoma
KICH	Kidney Chromophobe
KIRC	Kidney renal clear cell carcinoma
KIRP	Kidney renal papillary cell carcinoma
LAML	Acute Myeloid Leukemia
LGG	Lower Grade Glioma
LIHC	Liver hepatocellular carcinoma
LUAD	Lung adenocarcinoma
LUSC	Lung squamous cell carcinoma
MESO	Mesothelioma
OV	Ovarian serous cystadenocarcinoma
PAAD	Ovarian serous cystadenocarcinoma
PCPG	Pheochromocytoma and Paraganglioma
PRAD	Prostate adenocarcinoma
READ	Rectum adenocarcinoma
SARC	Sarcoma
SKCM	Skin Cutaneous Melanoma
STAD	Stomach adenocarcinoma
TGCT	Testicular Germ Cell Tumor

THCA	Thyroid carcinoma
THYM	Thymoma
UCEC	Uterine Corpus Endometrial Carcinoma
UCS	Uterine Carcinosarcoma
UVM	Uveal Melanoma

2. Figure S1. A sketch for the Transwell assay.

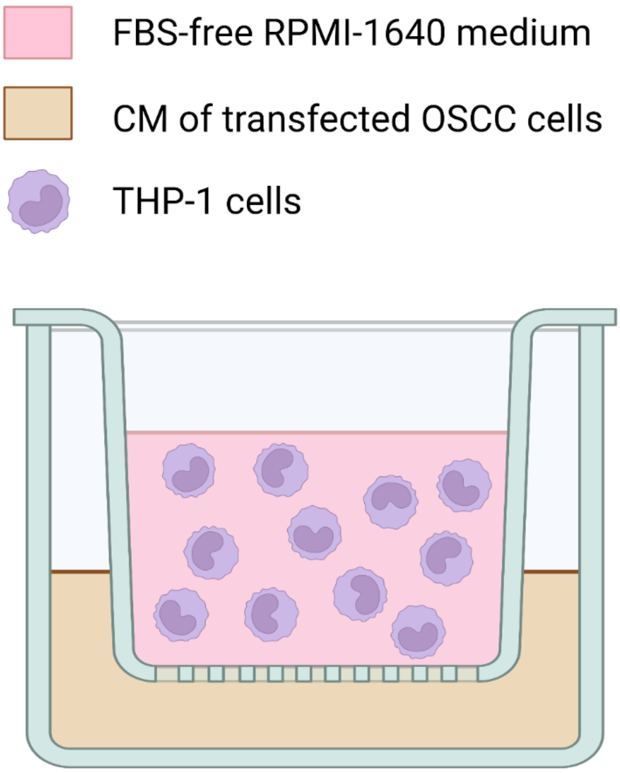

3. Figure S2. Correlation between CCR7 and CD68.

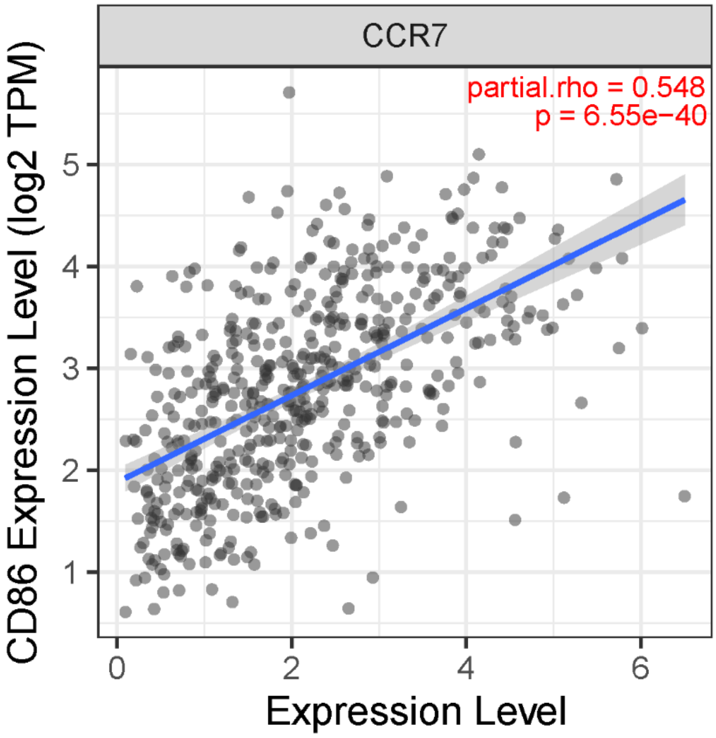

4. Figure S3. mRNA expression level of CD68 in M0 macrophages.

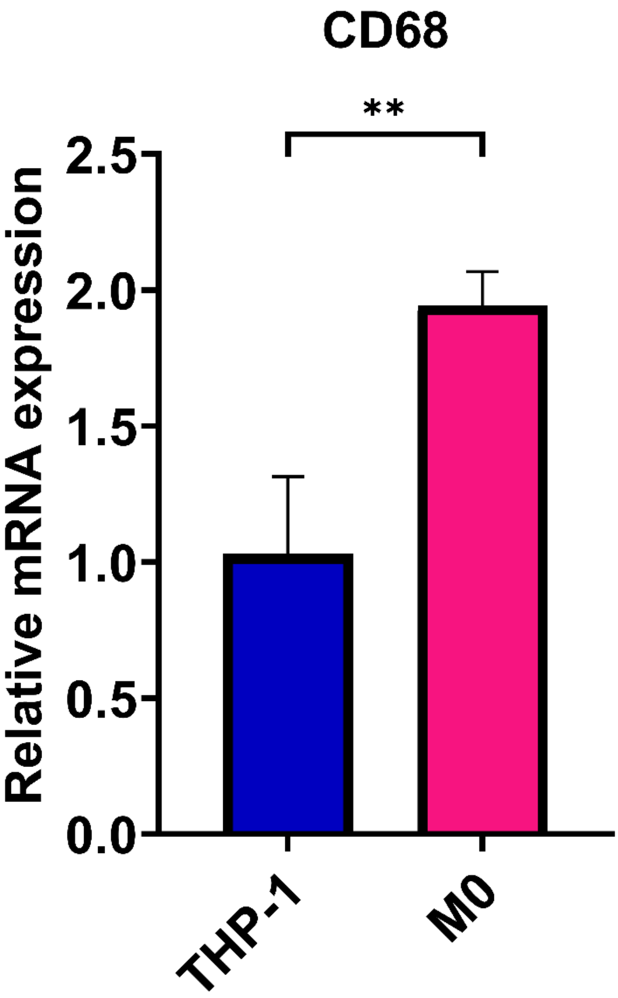

5. Table S4. A sketch for the M0 macrophages and OSCC cells coculture system.

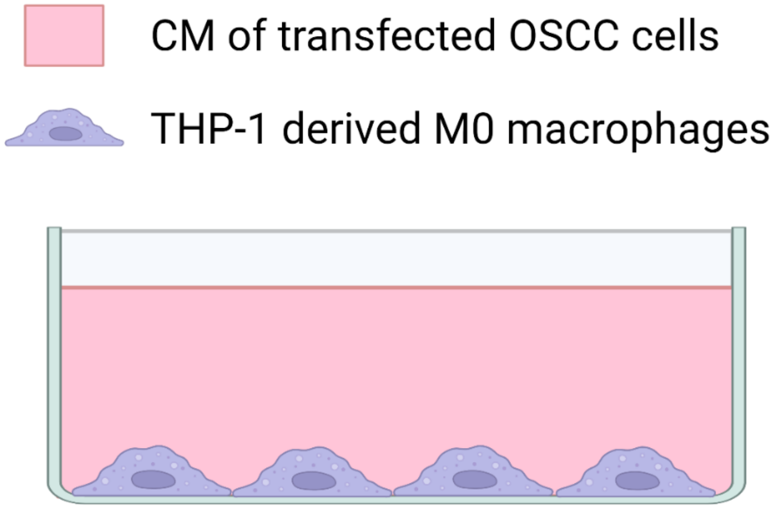

Supplement: Supplementary file 1 — Supplementary file1 (PDF 467 kb) [file 12672_2022_533_MOESM1_ESM.pdf]
